# Rap1-mediated nuclear factor-kappaB (NF-*κ*B) activity regulates the paracrine capacity of mesenchymal stem cells in heart repair following infarction

**DOI:** 10.1038/cddiscovery.2015.7

**Published:** 2015-08-24

**Authors:** Y Zhang, S Chiu, X Liang, F Gao, Z Zhang, S Liao, Y Liang, Y-H Chai, D J H Low, H-F Tse, V Tergaonkar, Q Lian

**Affiliations:** 1 Division of Cardiology, Department of Medicine, The University of Hong Kong, Hong Kong SAR, China; 2 Research Centre of Heart, Brain, Hormone, and Healthy Aging, Li Ka Shing Faculty of Medicine, The University of Hong Kong, Hong Kong SAR, China; 3 Department of Ophthalmology, Li Ka Shing Faculty of Medicine, The University of Hong Kong, Hong Kong SAR, China; 4 Institute of Molecular and Cellular Biology, Biopolis, Singapore; 5 Shenzhen University Health Science Center, Shenzhen, China

## Abstract

Paracrine effect is the major mechanism that underlies mesenchymal stem cells (MSC)-based therapy. This study aimed to examine how Rap1, telomeric repeat-binding factor 2-interacting protein 1 (Terf2IP), which is a novel modulator involved in the nuclear factor-kappaB (NF-*κ*B) pathway, regulates the paracrine effects of MSC-mediated heart repair following infarction. NF-*κ*B activity of stromal cells was increased by Rap1 as measured by pNF-*κ*B-luciferase reporter activity, and this was abolished by IkB-dominant-negative protein. Knockdown of Rap1 with shRap1 resulted in diminished translocation of p65-NF-*κ*B from the cytoplasm to nuclei in response to tumor necrosis factor-*α* (TNF-*α*) stimulation. Compared with BM-MSCs, Rap1^−/−^-BM-MSCs displayed a significantly reduced ratio of phosphorylated NF-*κ*B to NF-*κ*B-p65 and of Bax to Bcl-2, and increased resistance to hypoxia-induced apoptosis by the terminal deoxynucleotidal transferase-mediated dUTP nick end labeling (TUNEL) assay. In contrast, re-expression of Rap1 in Rap1^−/−^-BM-MSCs resulted in loss of resistance to apoptosis in the presence of hypoxia. Moreover, absence of Rap1 in BM-MSCs led to downregulation of NF-*κ*B activity accompanied by reduced pro-inflammatory paracrine cytokines TNF-*α*, IL (interleukin)-6 and monocyte chemotactic protein-1 in Rap1^−/−^-BM-MSCs compared with BM-MSCs. The apoptosis of neonatal cardiomyocytes (NCMCs) induced by hypoxia was significantly reduced when cocultured with Rap1^−/−^-BM-MSC hypoxic-conditioned medium (CdM). The increased cardioprotective effects of Rap1^−/−^-BM-MSCs were reduced when Rap1^−/−^-BM-MSCs were reconstituted with Rap1 re-expression. Furthermore, *in vivo* study showed that transplantation of Rap1^−/−^-BM-MSCs significantly improved heart function, decreased infarct size, prevented cardiomyocyte apoptosis and inhibited inflammation compared with controls and BM-MSCs (*P*<0.01). This study reveals that Rap1 has a critical role in the regulation of MSC paracrine actions. Compared with BM-MSCs, Rap1^−/−^-BM-MSCs decreased NF-*κ*B sensitivity to stress-induced pro-inflammatory cytokine production and reduced apoptosis. Selective inhibition of Rap1 in BM-MSCs may be a novel strategy to enhance MSC-based therapeutic efficacy in myocardial infarction.

## Introduction

Recent developments in stem cell biology to prevent or treat heart failure have moved from experimental research to clinical trials using different types of adult stem cells, such as adult bone marrow stem cells or mesenchymal stem cells (MSCs).^1,2^ Despite the promising results observed in preclinical and clinical studies, the potential mechanisms underlying stem cell-based therapy have not been fully classified. Accumulating evidence demonstrates that apart from transdifferentiation, it is the paracrine effects of MSCs that are predominately responsible for cardiac repair.^3^ Indeed, the exact extent to which these cells transdifferentiate into new cardiomyocytes to improve heart function remains highly controversial.^4,5^ There is increasing recognition that MSCs produce a variety of cytokines that can directly promote cell survival and regulate inflammation following myocardial infarction (MI).^3,6^ Various cytokines and growth factors have been investigated in different tissue-derived MSCs.^7,8^ Some cytokines and growth factors have been shown to be critical for cardiac protection, for example, basic fibroblast growth factor, vascular endothelial growth factor, stromal-derived factor,^9,10^ secreted frizzled related protein,^11^ interleukin-10 (IL-10)^12^ and metalloproteinase-9.^13^ In contrast, some cytokines and factors produced from MSCs, including tumor necrosis factor-*α* (TNF-*α*) and interleukin-6 (IL-6),^14^ are harmful for heart recovery. As a result, injection of MSCs or total MSC paracrine factors without optimization may limit their therapeutic efficacy. Therefore, optimization of MSCs before transplantation to maximize cell survival and beneficial paracrine factors is important. Nevertheless, the potential mechanism underlying regulation of MSC secretion is poorly understood and identification of the key molecules that govern MSC secretion to protect against heart ischemia injury remains urgently to be addressed.

Rap1 (Trf2IP), a telomeric repeat-binding factor 2-interacting protein 1 (Terf2IP), is a novel modulator involved in the Nuclear factor-kappaB (NF-*κ*B) pathway.^15,16^ Many telomeric proteins apart from Rap1 including telomerase are now known to regulate inflammation through NF-*κ*B signaling.^17,18^ It has been documented that NF-*κ*B family factors have important roles in the regulation of mitochondrial ROS/bioenergy, DNA replication, cell survival and inflammation in many cell types,^19–22^ including MSCs.^23^ Prior studies have also demonstrated that NF-*κ*B activation mediates cytokine/growth factor secretion by MSCs.^24^ Given the multifaceted effects of NF-*κ*B activity on MSCs, efforts to identify important regulators (s) that modulate specificity in the functioning of the NF-*κ*B signal pathway is a major challenge. Use of a genetic engineering will provide a greater understanding of the roles and mechanisms of specific factors that modulate NF-*κ*B activation in MSC-mediated cardioprotection. It also has been reported that MSCs can secrete a variety of cytokines to regulate inflammation and enable cardiac repair posttransplantation.^25^ Hence, to determine whether Rap1 can regulate MSC paracrine factors, which thereby impinges on cardiac repair, we designed this study. In this study, we proposed that inhibition of Rap1 can decrease pro-inflammatory factors secreted by MSCs, enhance cell survival and thus improve its therapeutic effects in MI. In this study, we showed that Rap1^−/−^-BM-MSCs have a better therapeutic efficacy than wild-type (WT) BM-MSCs for cardiac repair post MI in mice. The greater therapeutic potential of Rap1^−/−^-BM-MSCs for cardiac repair is not only attributed to the higher cell survival post-transplantation, but also to a reduced secretion of pro-inflammatory cytokines. Our results may prompt the development of new therapeutic strategies to enhance MSC-based therapy in MI.

## Results

### Characterization of BM-MSCs and Rap1^−/−^-BM-MSCs

**Figure 1:**
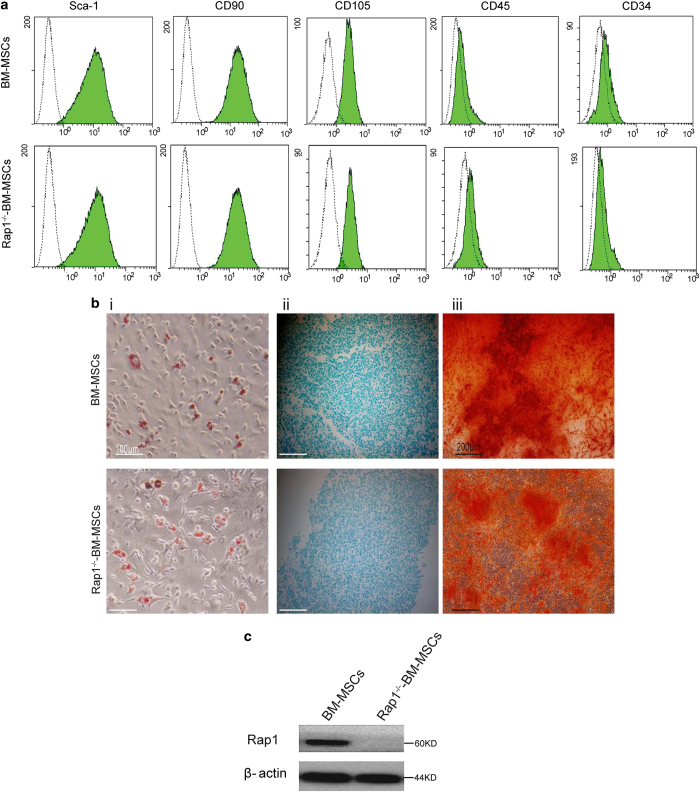
Characterization of BM-MSCs and Rap1^−/−^-BM-MSCs. (**a**) Surface markers profiling determined with FASC that is positive for Sca-1, CD90 and CD105; negative for CD34 and CD45. (**b**) (i) Oil red staining for adipogenesis. (ii) Alcian blue staining for chondrogenesis. (iii) Alizarin red staining for osteogenesis. (**c**) Expression of Rap1 in BM-MSCs and Rap1^−/−^-BM-MSCs.

As shown in Figure 1a, FACS analysis demonstrated that control BM-MSCs and Rap1^−/−^-BM-MSCs have similar surface markers that are defined in BM-MSCs, that is, Sca (+), CD90 (+), CD105 (+), CD45 (−) and CD34 (−; Figure 1a). Oil red staining showed that adipogenesis was highly observed in >80% of BM-MSCs and Rap1^−/−^-BM-MSCs (Figure 1bi). Alcian Blue staining showed that chondrogenesis was efficient with >80% cells for calcium deposition (Figure 1bii). Alizarin Red staining revealed that BM-MSCs and Rap1^−/−^-BM-MSCs could differentiate into osteogenic cells (Figure 1biii). In addition, western blot analysis showed that Rap1 was detected in BM-MSCs, but not in Rap1^−/−^-BM-MSCs (Figure 1c), indicating the total deletion of Rap1 from Rap1^−/−^-BM-MSCs.

### Activation of NF-*κ*B transcriptional activity by Rap1

**Figure 2:**
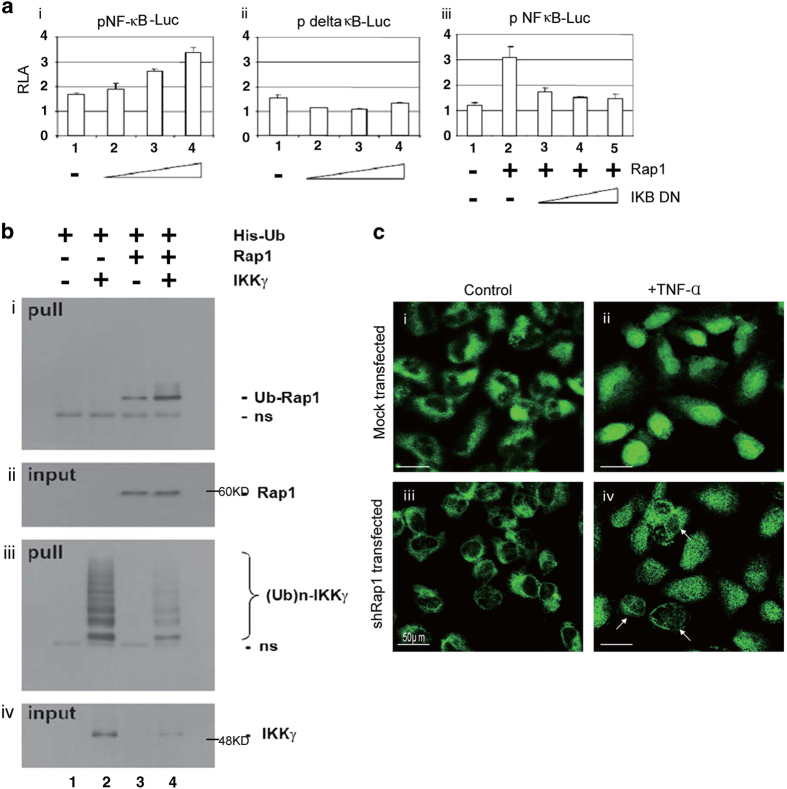
Activation of NF-*κ*B transcriptional activity by Rap1. (**a**) HeLa cells were transfected with indicated plasmids. Groups i and iii received WT reporter constructs p NF-*κ*B-Luc, whereas group B received delta NF-*κ*B-Luc. All groups received pSV-RLuc. The amounts of expression plasmid for Rap1 (i and ii) and IkB-dominant-negative (iii) were progressively increased as indicated. Relative luciferase activity (RLA) represents firefly luciferase activity recovered from pNF-*κ*B-Luc or p delta *κ*B-Luc normalized to Renilla luciferase activity recovered from pSV-RLuc. (**b**) HEK293T cells were transfected with expression plasmids for Flag-tagged Rap1 (lanes 3 and 4) and HA-tagged IKK*γ* (lanes 2 and 4). All groups received His-tagged ubiquitin expression plasmid. His-tagged ubiquitin conjugates were pulled down. The ubiquitinated Rap1 and IKK*γ* was detected using western blot analysis with anti-Flag (i) and anti-HA (ii), respectively. The input lysates were also probed for anti-Flag and anti-HA to detect Rap1 and IKK*γ*, respectively (ii and iv). (**c**) Representative photographs show the location of NF-*κ*B-p65 in HeLa cells (i) and shRap1-transfected HeLa cells (iii). NF-*κ*B-p65 was translocated from the cytoplasm to nuclei responding to TNF stimulation (ii). Knocking down Rap1 with shRap1 resulted in diminished translocation of NF-*κ*B-p65 from the cytoplasm to nuclei responding to TNF stimulation (iv).

In order to address the direct relationship between NF-*κ*B and Rap1, the activation of NF-*κ*B transcriptional activity by Rap1 was examined. A reporter gene construct carrying NF-*κ*B-Luc was used to report NF-*κ*B activity. This construct contains an NF-*κ*B enhancer element located upstream of the secreted luciferase gene. Binding of transcription factors to the NF-*κ*B enhancer element allows luciferase (tLuc) to be expressed and secreted into the surrounding medium (Catalog No. 631743; Clontech Laboratories, Mountain View, CA, USA). As shown in Figure 2a, HeLa cells were transfected with indicated plasmids. Groups i and iii received WT reporter constructs p NF-*κ*B-Luc, whereas group ii received delta NF-*κ*B-Luc. All groups received pSV-RLuc. The amount of expression plasmid for Rap1 (i and ii) and IkB-dominant-negative (iii) was progressively increased as indicated. Relative luciferase activity represents firefly luciferase activity recovered from pNF-*κ*B-Luc or pdelta *κ*B-Luc normalized to Renilla luciferase activity recovered from pSV-RLuc (Figure 2a). The results presented showed that the activity of NF-*κ*B transcriptional activity was activated by Rap1.

Next, the relationship of IKK*γ* and Rap1 was examined. As shown in Figure 2b, HEK293T cells were transfected with expression plasmids for Flag-tagged Rap1 (lanes 3 and 4), HA-tagged IKK*γ* (lanes 2 and 4). All groups received His-tagged ubiquitin expression plasmid. His-tagged ubiquitin conjugates were pulled down. The ubiquitinated Rap1 and IKK*γ* were detected using western blot analysis with anti-Flag (Figure 2bi) and anti-HA (Figure 2biii), respectively. The input lysates were also probed for anti-Flag and anti-HA to detect Rap1 and IKK*γ*, respectively (Figure 2bii and iv). These results show the IKK*γ*-mediated ubiquitination of Rap1.

Immunostaining results demonstrated that NF-*κ*B translocated from the cytoplasm to nuclei when HeLa cells were stimulated with TNF-*α* (Figure 2ci and ii). Nonetheless, translocation of NF-*κ*B-p65 from the cytoplasm to nuclei responding to TNF-a stimulation was diminished by knocking down Rap1 with shRap1 (Figure 2ciii and iv). These results show that the absence of Rap1 negatively regulates activation of NF-*κ*B.

### Rap1^−/−^-BM-MSCs are more tolerant than BM-MSCs to hypoxia-induced apoptosis

**Figure 3:**
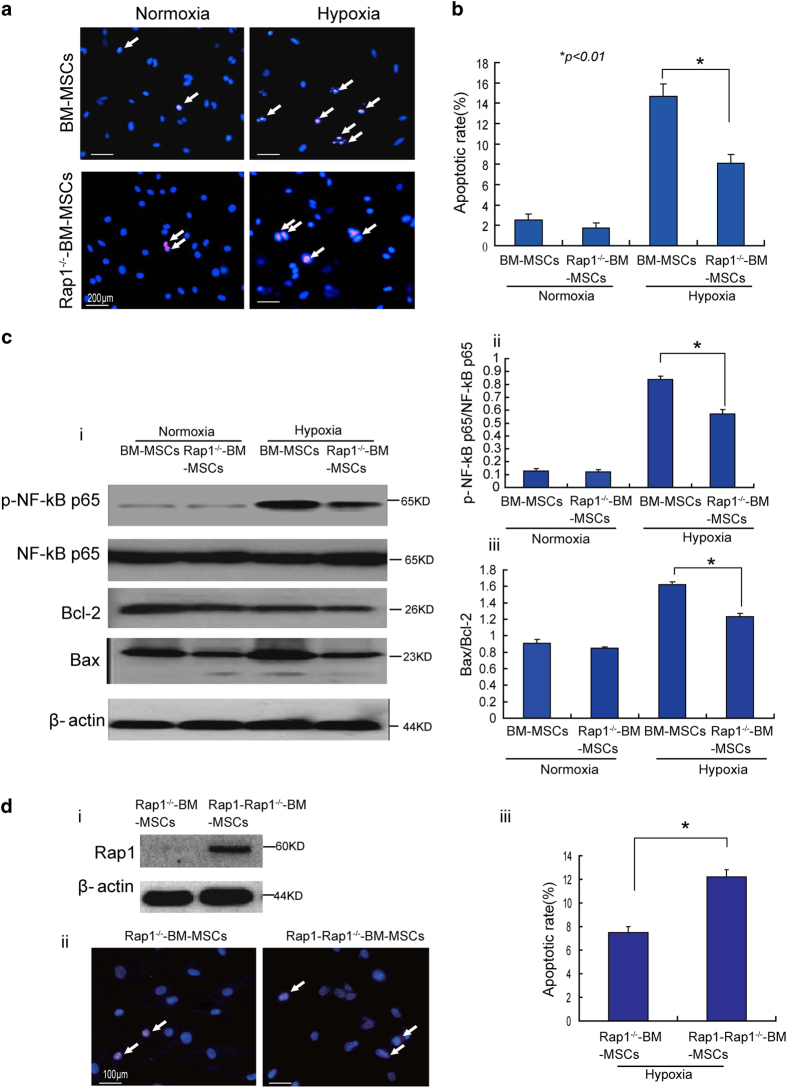
Rap1^−/−^-BM-MSCs are more tolerant than BM-MSCs against hypoxia challenge. (**a**) Representative images show apoptosis of BM-MSCs and Rap1^−/−^-BM-MSCs under normoxia and hypoxia. (**b**) The apoptotic rate was quantitatively measured in BM-MSCs and Rap1^−/−^-BM-MSCs under normoxia and hypoxia (*P<0.01). (**c**) (i) Western blot analysis shows the expression of p-p65-NF-*κ*B, p65-NF-*κ*B, Bcl-2 and Bax of BM-MSCs and Rap1^−/−^-BM-MSCs under normoxia and hypoxia. (ii) Quantitative measurement of pNF-*κ*B-p65/NF-*κ*B-p65 of BM-MSCs and Rap1^−/−^-BM-MSCs under normoxia and hypoxia after normalized to *β*-actin (*P<0.01). (iii) Quantitative measurement of Bax/Bcl-2 of BM-MSCs and Rap1^−/−^-BM-MSCs under normoxia and hypoxia (*P<0.01). (**d**) (i) Western blot analysis shows overexpressed Rap1 in Rap1^−/−^-BM-MSCs. (ii) Representative images show apoptosis of Rap1^−/−^-BM-MSCs and Rap1-Rap1^−/−^-BM-MSCs under hypoxia. White arrows denote apoptotic cells. (iii) The apoptotic rate was quantitatively measured in Rap1^−/−^-BM-MSCs and Rap1-Rap1^−/−^-BM-MSCs under hypoxia (*P<0.01). All graphs represent the mean and error bars represent S.D. Data are representative of at least three independent experiments.

Apoptosis of Rap1^−/−^-BM-MSCs and BM-MSCs under normoxic or hypoxic conditions was examined. As shown in Figures 3a and b, the apoptotic rate of Rap1^−/−^-BM-MSCs and BM-MSCs under normoxia was similar and very low (Figure 3b, 2.55±0.59% *versus* 1.7±0.52%; *P*>0.05). Nonetheless, compared with normoxia, the apoptotic rate was significantly increased under hypoxic conditions. Notably, compared with BM-MSCs, the apoptotic rate of Rap1^−/−^-BM-MSCs under hypoxia was much lower (Figure 3b, 17.7±1.2% *versus* 8.1±0.87%; *P*<0.01), suggesting that Rap1^−/−^-BM-MSCs have superior resistance to the challenge of hypoxia.

As shown in Figure 3c, western blot analysis results demonstrated that hypoxia significantly increased the ratio of p-NF-*κ*B/NF-*κ*B-p65 and the ratio of Bax/Bcl-2 in Rap1^−/−^-BM-MSCs and BM-MSCs. Notably, compared with BM-MSCs, NF-*κ*B activation and the levels of Bax/Bcl-2 were greatly reduced in Rap1^−/−^-BM-MSCs under conditions of hypoxia (Figure 3c).

To further understand the role of Rap1 in the regulation of MSCs against hypoxia, deficiency of Rap1^−/−^-BM-MSCs was replenished with Rap1 re-expression using a lentivirus system. As shown in Figure 3di, Rap1 was detected in Rap1-Rap1^−/−^-BM-MSCs but not in Rap1^−/−^-BM-MSCs. Under hypoxic challenge, apoptosis of Rap1-Rap1^−/−^-BM-MSCs was much higher than Rap1^−/−^-BM-MSCs (Figure 3dii and iii; *P*<0.01). These results further confirm that deletion of Rap1 increases the resistance of MSCs to the challenge of hypoxia.

### CdM of Rap1^−/−^-BM-MSCs alleviates hypoxia-induced NCMC apoptosis

**Figure 4:**
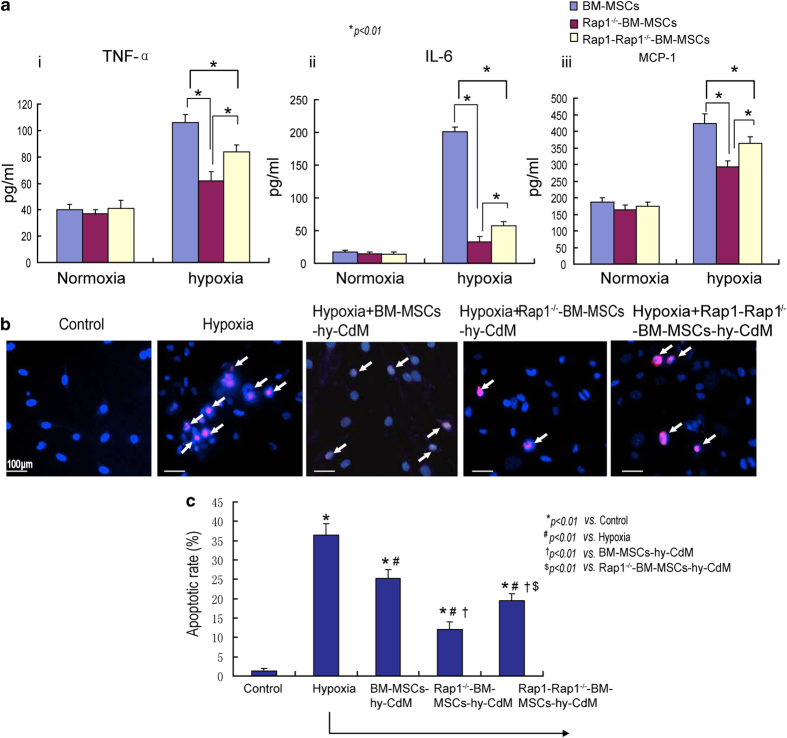
Distinct paracrine effects of BM-MSCs, Rap1^−/−^-BM-MSCs and Rap1-Rap1^−/−^-BM-MSCs. (**a**) The expression of TNF-*α*, IL-6 and MCP-1 of BM-MSCs, Rap1^−/−^-BM-MSCs and Rap1-Rap1^−/−^-BM-MSCs under normoxia and hypoxia (*P<0.01). (**b**) Representative images show apoptosis of NCMCs cocultured with the hypoxia-CdM from BM-MSCs, Rap1^−/−^-BM-MSCs and Rap1-Rap1^−/−^-BM-MSCs under hypoxic condition. White arrows denote the apoptotic cells. (**c**) The apoptotic rate of NCMCs was measured among the different experimental groups (*P<0.01 *versus* the control group; ^*#*^ *P<*0.01 *versus* the hypoxia group; ^†^ *P*<0.01 *versus* the BM-MSCs-hy-CdM group; ^$^ *P*<0.01 *versus* the Rap1^−/−^-BM-MSCs-hy-CdM group). All graphs represent the mean and error bars represent S.D. Data are representative of at least three independent experiments.

The pro-inflammatory factors from Rap1^−/−^-BM-MSCs and BM-MSC-CdM under normoxic and hypoxic conditions were examined. As shown in Figure 4ai, ii and iii, compared with the normoxia-CdM (conditioned medium), the concentration of TNF-*α*, IL-6 and monocyte chemotactic protein 1 (MCP-1) was increased in hypoxia-CdM of Rap1^−/−^-BM-MSCs and BM-MSCs. Furthermore, in the hypoxia-CdM, the concentration of TNF-*α*, IL-6 and MCP-1 was much lower in the Rap1^−/−^-BM-MSC group than in the BM-MSC group (Figure 4a, *P*<0.01). Notably, the concentration of TNF-*α*, IL-6 and MCP-1 was also increased in Rap1-Rap1^−/−^-BM-MSC-hy-CdM compared with Rap1^−/−^-BM-MSC-hy-CdM, indicating that deletion of Rap1 reduces pro-inflammatory factor release by MSCs (Figure 4a).

Next, in order to examine the protective effects of MSC-hypoxia-CdM, neonatal cardiomyocytes (NCMCs) were divided into five groups and received the following different treatments: (1) control group; (2) 50 *μ*l serum- and antibiotic-free DMEM (Dulbecco’s modified Eagle’s medium; hypoxia group); (3) 50 *μ*l BM-MSCs hypoxia-CdM (BM-MSC-hy-CdM group); (4) 50 *μ*l Rap1^−/−^-BM-MSC hypoxia-CdM (Rap1^−/−^-BM-MSC-hy-CdM group); and (5) Rap1-Rap1^−/−^-BM-MSC hypoxia-CdM (Rap1-Rap1^−/−^-BM-MSC-hy-CdM group) and then cultured for 48 h under hypoxia. Then terminal deoxynucleotidal transferase-mediated dUTP nick end labeling (TUNEL) assay was performed to examine apoptosis in the different groups. As shown in Figures 4b and c, the apoptotic rate of NCMCs in the hypoxia group was dramatically increased compared with the control group (*P*<0.01). In contrast, the apoptotic rate in Rap1^−/−^-BM-MSC-hy-CdM-, BM-MSC-hy-CdM- and Rap1-Rap1^−/−^-BM-MSC-hy-CdM-treated groups was significantly decreased compared with the hypoxia group (*P*<0.01). In addition, the apoptotic rate in the Rap1^−/−^-BM-MSC-hypoxia-CdM-treated group was lower than that in the BM-MSC-hypoxia-CdM-treated group (*P*<0.01). Notably, the apoptotic rate was significantly increased in the Rap1-Rap1^−/−^-BM-MSC-hy-CdM group compared with the Rap1^−/−^-BM-MSC-hy-CdM group, indicating that increased Rap1 can downregulate the cardioprotection of Rap1^−/−^-BM-MSCs by regulating the paracrine effects (Figures 4b and c).

### Transplantation of Rap1^−/−^-BM-MSCs enhances cell survival and cardioprotection in MI

**Figure 5:**
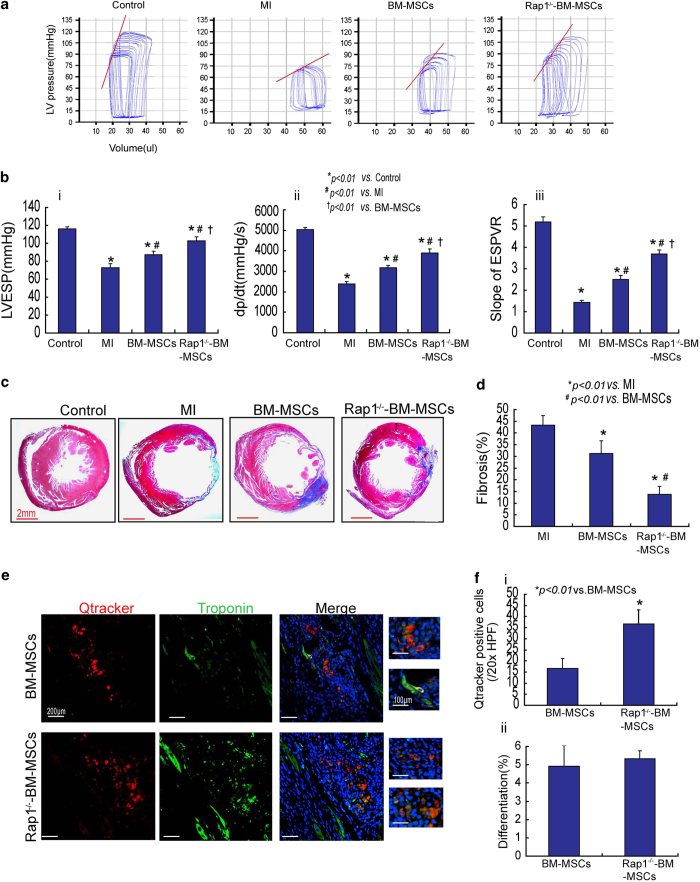
Rap1^−/−^-BM-MSC transplantation restores heart function, reduces infarction size and increases cell survival in mice of the MI group. (**a**) Cardiac catheterization shows BM-MSC or Rap1^−/−^-BM-MSC transplantation-restored LV function at 4 weeks after cell transplantation. (**b**) LVESP (i), +dp/dt (ii) and slope of ESPVR (iii) were quantitatively measured among the different experimental groups (*P<0.01 *versus* the control group; ^*#*^ *P<*0.01 *versus* the MI group; ^†^ *P*<0.01 *versus* the BM-MSC group). (**c**) Representative images show at 4 weeks post-cell transplantation, MSC transplantation reduced the infarction size. (**d**) Fibrosis formation was measured in heart tissue among different experimental groups (*P<0.01 versus the MI group; ^*#*^ *P*<0.01 *versus* BM-MSC group). (**e**) At 4 weeks post-cell transplantation, Qtracker-positive cells in the heart tissue were detected in BM-MSC or Rap1^−/−^-BM-MSC group. Moreover, some Qtracker-positive cells also coexpressed troponin, suggesting some grafted MSCs differentiated into cardiomyocytes. (**f**) (i) Qtracker-positive cell in the heart tissue was quantitatively measured in BM-MSC- or Rap1^−/−^-BM-MSC-treated mice (*P<0.01 *versus* BM-MSC group). (ii) The ratio of Qtracker-positive and troponin-positive cells to Qtracker-positive cell as percent of cell differentiation in BM-MSC- or Rap1^−/−^-BM-MSC-treated mice was calculated. All graphs represent the mean and error bars represent S.D. Data are representative of at least three independent experiments.

Invasive hemodynamic measurement at 4 weeks post-cell transplantation showed that, compared with the MI group, left ventricle end systolic pressure (LVESP), maximum dp/dt and slope of end systolic pressure volume relationship (ESPVR) were significantly increased (Figures 5a and b; *P*<0.01) in the MSC transplantation groups. Furthermore, LVESP, maximum dp/dt and slope of ESPVR were significantly higher in the Rap1^−/−^-BM-MSCs group than in the BM-MSCs group (Figures 5a and bi, ii and iii; *P*<0.01).

Cardiac fibrosis was assessed using Masson Trichrome staining (Figure 5c). At 4 weeks post-MSC transplantation, cardiac fibrosis was significantly reduced by 28.1% in the BM-MSCs group and by 68.2% in the Rap1^−/−^-BM-MSCs group, compared with the MI group (Figure 5d; *P*<0.01). Compared with the BM-MSCs group, the extent of cardiac fibrosis was also significantly lower in the Rap1^−/−^-BM-MSCs group (Figure 5d, 31.2±5.4% *versus* 13.8±3.3%; *P*<0.01).

Before cell transplantation, MSCs were labeled with Qtracker. The engrafted MSCs at 4 weeks post-transplantation in the ischemic myocardium were visualized and quantified. Both BM-MSCs and Rap1^−/−^-BM-MSCs were successfully engrafted in the ischemic heart tissue (Figure 5e). Notably, the number of Qtracker-positive cells that survived in the ischemic myocardium 4 weeks posttransplantation was much higher in the Rap1^−/−^-BM-MSCs group than in the BM-MSCs group (Figure 5fi, 36.6±6.4/20× *versus* 16.6±4.5/20×; *P<*0.01). Immunostaining with troponin showed that some Qtracker-positive cells in the ischemic myocardium differentiated into cardiomyocytes (Figure 5e). Nevertheless, only a small proportion of retained BM-MSCs (~4.9%) and Rap1^−/−^-BM-MSCs (~5.3%) had transdifferentiation or cell fusion to cardiomyocytes (Figure 5f ii).

### Rap1^−/−^-BM-MSCs transplantation reduces cardiomyocyte apoptosis linked to increased angiogenesis and decreased inflammation *in vivo*

**Figure 6:**
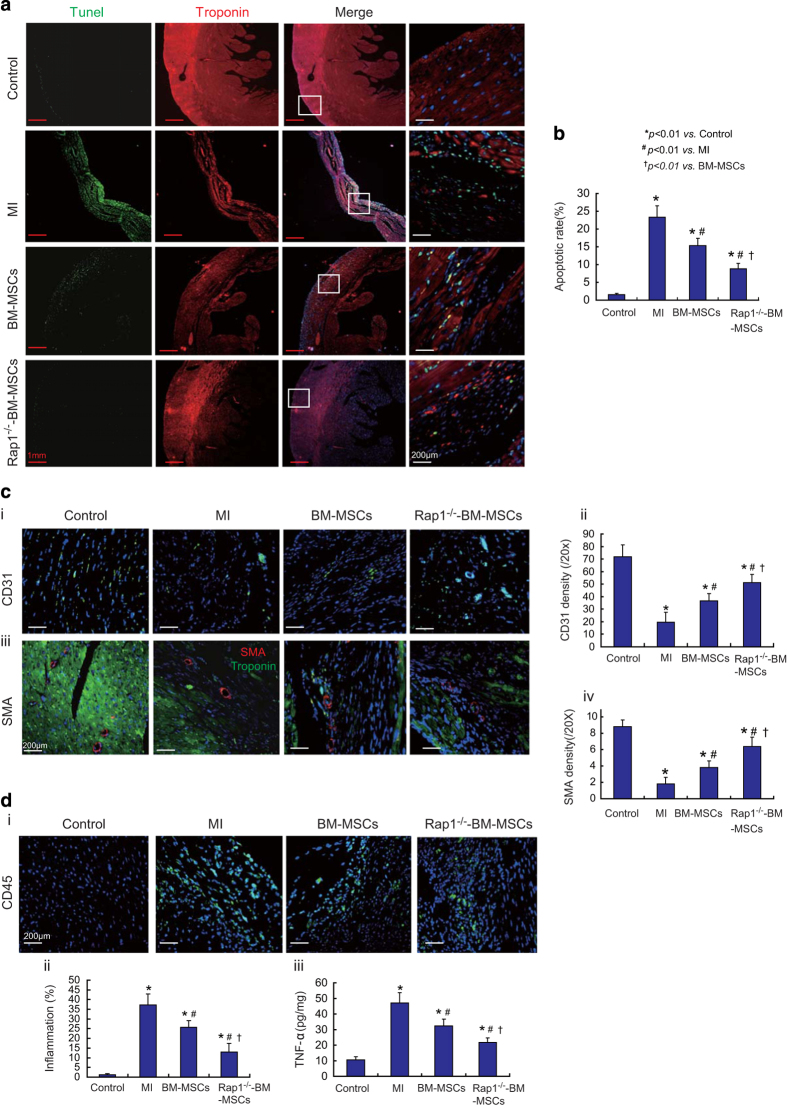
Rap1^−/−^-BM-MSC transplantation ameliorates cardiomyocyte apoptosis, enhances angiogenesis and reduces inflammation in the mice of the MI group. (**a**) Representative images show apoptosis of cardiomyocytes at 4 weeks post-cell transplantation in the heart tissue of MI. (**b**) The apoptotic rate was measured in heart tissue of different experimental groups. (**c**) Representative images show the capillaries (i) and blood vessels (iii) in the heart tissue at 4 weeks post-cell transplantation. (ii) The density of CD31 was measured in heart tissue among different experimental groups. (iv) The blood vessels in the heart tissue among the different groups were measured (*P<0.01 *versus* the control group; ^*#*^ *P<*0.01 *versus* the MI group; ^†^ *P<*0.01 *versus* the BM-MSC group). (**d**) (i) Representative images show inflammatory infiltration mononuclearcyte CD45 cells accumulated in the heart tissue among different groups. (ii) The inflammation in the different groups was measured as percent of CD45-positive staining cells *versus* DAPI-positive cells per myocardium area. (iii) The concentration of TNF-*α* among different groups was measured (*P<0.01 *versus* the control group; ^*#*^ *P<*0.01 *versus* the MI group; ^†^ *P*<0.01 *versus* the BM-MSCs group). All graphs represent the mean and error bars represent S.D. Data are representative of at least three independent experiments.

As shown in Figures 6a and b, compared with the control group, apoptosis of cardiomyocytes was significantly increased in the MI group (Figures 6a and b, 23.3±3.3% *versus* 1.5±0.4%; *P*<0.01). Transplantation of BM-MSCs or Rap1^−/−^-BM-MSCs significantly decreased the cardiomyocyte apoptotic rate (Figures 6a and b; *P*<0.01). In addition, the apoptotic rate in the Rap1^−/−^-BM-MSC group was much lower than in the BM-MSC group (Figures 6a and b, 15.3±2.1% *versus* 8.8±1.5%; *P<*0.01).

The capillary density of the MI area was evaluated by PECAM (CD31) staining. Compared with the control group, the capillary density was dramatically reduced in the MI group (Figure 6ci and ii, 71.7±9.7/20× *versus* 19.6±7.9/20×; *P<*0.01). Transplantation of BM-MSCs or Rap1^−/−^-BM-MSCs significantly increased the capillary density of the MI area (Figure 6ci and ii; *P*<0.01), and the capillary density in the Rap1^−/−^-BM-MSC group was much higher than in the BM-MSC group (Figure 6ci and ii, 51±6.6/20× *versus* 36.7±5.8%; *P<*0.01).

The blood vessels of the MI area were evaluated by smooth muscle actin (SMA) staining; blood vessels were greatly increased in the BM-MSC and Rap1^−/−^-BM-MSC groups compared with the MI group (Figure 6ciii and iv; *P*<0.01). Moreover, blood vessel density was much higher in the Rap1^−/−^-BM-MSC group than in the BM-MSC group (Figure 6ciii and iv, 6.4±1.1/20× *versus* 3.8±0.8/20×; *P<*0.01).

One week post-cell transplantation, inflammation in heart tissue among the different experimental groups was evaluated by CD45 staining and ELISA. As shown in Figure 6di and ii, inflammation in the MI group was dramatically increased compared with the control group (Figure 6di and ii, 37.3±5.6% *versus*1.2±0.6%; *P<*0.01). Transplantation of BM-MSCs or Rap1^−/−^-BM-MSCs significantly decreased this inflammation (Figure 6di and ii; *P*<0.01). The inflammation in the Rap1^−/−^-BM-MSC group was much lower than that in the BM-MSC group (Figure 6di and ii, 25.8±3.3% *versus* 13±4.5%; *P<*0.01).

Similar results were seen in the measurement of TNF-*α* using ELISA in the different groups. Nonetheless, as shown in Figure 6diii, 1 week following MSC transplantation, TNF-*α* was markedly reduced by 31.3 and 53.6% in the BM-MSC and Rap1^−/−^-BM-MSCs groups, respectively, compared with the MI group (Figure 6d and iii; *P*<0.01). In addition, TNF-*α* in Rap1^−/−^-BM-MSCs was much lower than in BM-MSCs (Figure 6diii, 33.3±4.5 *versus* 21.8±2.7 pg/mg; *P<*0.01).

## Discussion

There are several major findings in this study. First, Rap1^−/−^-BM-MSCs were more tolerant than BM-MSCs to hypoxia that is associated with reduced activation of NF-*κ*B activity. Second, compared with BM-MSCs, Rap1^−/−^-BM-MSCs showed a better therapeutic efficacy in MI. Third, Rap1^−/−^-BM-MSC-mediated cardiac repair was associated with reduced inflammation post MI and enhanced cell survival. Finally, secretion profiling revealed reduced pro-inflammatory cytokines in Rap1^−/−^-BM-MSCs such as TNF-*α*, IL-6 and MCP-1.

NF-*κ*B has been documented as a pro- or anti-apoptotic gene that depends on the cellular context^26–28^ and which upstream signaling it is activated by. NF-*κ*B is also essential for the survival of many cells and functional response to stress.^23^ In endothelial cells, inhibition of NF-*κ*B activity significantly attenuates apoptosis induced by hypoxia.^29^ In this study, under hypoxic conditions, apoptosis of Rap1^−/−^-BM-MSCs was much lower than that of BM-MSCs because of inhibition of NF-*κ*B activity and Bax/Bcl-2, and was confirmed using the western blot analysis. The *Rap1* gene is a novel adaptor that regulates NF-*κ*B activity by serving as the adaptor of the IKK complex, having a critical role in regulating cell apoptosis^15^ and metabolism.^30^ In this study, we showed that absence of Rap1 negatively regulated the activation of NF-*κ*B in MSC. Our data also demonstrated that deletion of Rap1 in MSCs inhibits the activation of NF-*κ*B, reduces the ratio of Bax/Bcl-2 and then directly attenuates the apoptosis induced by hypoxia, suggesting that inhibition of Rap1 enhances MSC survival capacity under hypoxia. These results prompt us to consider Rap1^−/−^-BM-MSC transplantation instead of BM-MSCs for MI in that Rap1^−/−^-BM-MSCs have a stronger ability to survive in the hostile environment of the injured heart. In our *in vivo* study, we confirmed that 4 weeks after transplantation, more Rap1^−/−^-BM-MSCs than BM-MSCs survived and contributed more to the recovery of heart function.

NF-*κ*B activation enhances expression of pro-inflammatory cytokines such as IL-6, IL-8 and TNF-*α*, and then provokes an excessive inflammatory response, with detrimental consequences.^31,32^ Total inhibition of NF-*κ*B leads to cell functional defects.^33^ Selective inhibition of certain signals to reduce NF-*κ*B activity has been proposed as a novel therapeutic strategy in many inflammatory diseases and cancer treatment.^34,35^ Previous studies have revealed that MSCs have contradictive effects with pro- and anti-inflammatory properties.^36^ Enhancement of the anti-inflammatory effects of MSCs would be important to increase the efficacy of cardiovascular repair.^37,38^ Thus, deletion of Rap1 can regulate NF-*κ*B activation, thereby mediating pro- and anti-inflammatory cytokine release. Our data demonstrated that, under hypoxia, the expression of pro-inflammatory cytokines IL-6, TNF-*α* and MCP-1 in the Rap1^−/−^-BM-MSCs was much lower than in BM-MSC CdM. This suggests that the absence of Rap1 reduces NF-*κ*B activation and decreases the pro-inflammatory cytokine production by MSCs. Furthermore, hypoxia-CdM from Rap1^−/−^-BM-MSCs demonstrated a superior protective effect to BM-MSCs against myocardial death under hypoxia challenge, indicating that the beneficial paracrine function is enhanced in Rap1^−/−^-BM-MSCs compared with WT MSCs. To further confirm the key role of Rap1 in regulating pro-inflammatory cytokine release by MSCs, we transfected Rap1^−/−^-BM-MSCs with Rap1; when Rap1^−/−^-BM-MSCs overexpressed Rap1, the expression of IL-6, TNF-*α* and MCP-1 was elevated under hypoxic conditions, suggesting that deletion of Rap1 has a strong capacity to regulate the paracrine action of MSCs.

There are some aspects of this study that need further investigation. First, the secretion of MSCs in the ischemic heart may differ under *in vitro* hypoxia. Second, apart from the anti-inflammatory reaction of MSCs in this study, many other potential mechanisms of MSCs need to be further investigated. Third, whether deletion of Rap1 can influence other potential signaling pathways to mediate MSC-based therapy for cardiovascular disease also needs further investigation.

In summary, in this study we established that a novel NF-*κ*B adaptor, Rap1, has an important role in the regulation of MSC paracrine components and MSC capacity for survival in a stressful environment. Compared with WT BM-MSCs, transplantation of Rap1^−/−^-BM-MSCs achieved superior therapeutic efficacy in a mouse model of MI that may be attributed to Rap1-modulated cytokine bias and cell survival potential.

## Materials and Methods

### Mice

The WT and Rap1^−/−^ mice, provided by the National University of Singapore, were bred and kept in the Laboratory Animal Unit at the University of Hong Kong. All animal experiments in this study were approved by the Committee on the Use of Live Animals in Teaching and Research at The University of Hong Kong.

### Isolation, culture and characterization of Rap1^−/−^-BM-MSCs and BM-MSCs

Rap1^−/−^-BM-MSCs and BM-MSCs were isolated from 6~8-week-old Rap1^−/−^ and WT mice, respectively. Briefly, following killing, femurs and tibiae were quickly removed from mice and bone marrow was harvested by strong flushing with DMEM (Invitrogen) supplemented with 10% fetal bovine serum (FBS, Gibco, USA) and P/S on ice. The resultant cell suspension was filtered using a 70-mm filter and bone marrow cells were planted in culture dishes with complete DMEM medium and incubated at 37 °C with 5% CO_2_ in a humidified chamber. Non-adherent cells were removed 3 h later by replacing the culture medium. Thereafter, the culture medium was replaced with fresh medium and the process repeated every 8 h until 72 h of initial culture was reached. MSCs were characterized using flow cytometry. Oil red staining was performed to identify adipocytes, alcian blue staining for chondrocytes and alizarin red staining for osteocytes. In order to track MSCs posttransplantation, MSCs were labeled with the cell tracker dye Qtracker 655 Cell Labeling Kit (Q25021MP, Life Technologies, Carlsbad, CA, USA) according to the manufacturer’s instructions. The labeling efficiency was verified using flow cytometry.

### Preparation of CdM

The CdM of cultured Rap1^−/−^-BM-MSCs and BM-MSCs was collected as previously reported.^39^ Briefly, MSCs were rypsinized and plated on a 10-cm plate. After 24 h, the completed culture medium was replaced with serum- and antibiotic-free DMEM. Twenty-four hours later, serum-free cell culture supernatants were collected, filtered by a 0.22-*μ*m filter and centrifuged at 4 °C, 4000×*g* for 30 min using Amicon Ultra-4 Centrifugal Filter Devices (Millipore Billerica, MA, USA) to produce CdM. The final concentration was adjusted to 20× of collected CdM.

### NCMC isolation and culture

The NCMCs were isolated and cultured as described previously.^40^ Briefly, following killing of neonatal Wistar rats (0- to 1-day-old), the hearts were promptly removed, rinsed four times with modified Hank’s solution and cut into small pieces on ice. The tissue fragments were transferred to a 50-ml tube and warmed in a water bath with a magnetic bar for 10 min at 37 °C. After discarding the supernatant, the minced myocardium was digested with fresh pre-warmed 0.25% trypsin for 5 min at 37 °C, and the supernatant collected gently and transferred to a 50-ml tube on ice containing 7 ml FBS. These two steps were repeated to collect supernatant after which all supernatants were centrifuged at 156.8×*g* for 5 min to collect the cells. Cells were re-suspended in NRVM culture medium to reduce fibroblast contamination. Finally, the supernatant was aspirated gently, and the cells were plated in MEA dishes at a density of 6×0^5^ cells/ml. Culture medium was changed every day.

### TUNEL assay

To directly visualize apoptosis of MSCs exposed to hypoxia and the NCMCs cocultured with MSC-CdM under hypoxic conditions, TUNEL assay was performed according to the manufacturer’s instructions. Briefly, after washing with PBS, the cells on the cover slide were incubated with 1 *μ*g/ml Proteinase K/10 mM Tris solution at room temperature for 15 min and then washed twice in PBS and finally incubated with 50 *μ*l TUNEL reaction mixture in a humid chamber for 1 h at room temperature. Cover slides were washed twice with PBS, mounted with 4, 6-diamidino-2-phenylindole (DAPI), observed under a fluorescent microscope and finally photographed.

### Western blot analysis

Total proteins were extracted from cells using RIPA buffer and the concentration measured. Protein (20 *μ*g) was separated in a 10% SDS-PAGE gel and transferred on a PVDF membrane (Millipore). The membrane was washed three times in Tris-buffered saline (TBS) with 0.1% Tween-20 and blocked with 5% fat-free milk in TBS for 1 h at room temperature. Subsequently, the membrane was incubated with anti-Rap1, Bcl-2, Bax, p-NF-*κ*B-p65 and NF-*κ*B-p65 (Santa Cruz, Dallas, TX, USA; 1 : 2000) overnight at 4 °C followed by incubation with horseradish peroxidase-conjugated secondary antibodies (1 : 10 000 dilution; Santa Cruz). Finally, the signal on the membranes was visualized using ECL and exposed to medical X-ray films for seconds or minutes.

### Lentiviral construct packaging and infection

The lentivirus was packaged by co-transfecting 293T cells using the lentiviral packaging system comprising the recombinant lentiviral transfer Rap1 plasmid or shRap1 plasmid, packaging (GAG/Pol and REV) plasmids and envelope (VSV-G) plasmid. Following 48-h culture, the supernatant of transfecting 293T cells was collected, concentrated and tittered. Subsequently, the virus was used to infect Rap1^−/−^-BM-MSCs or HeLa cells.

### MI model and cell transplantation

A MI model was induced in ICR mice (6~8-week-old). Mice were anesthetized with an intraperitoneal injection of ketamine (100 mg/kg) and xylazine (20 mg/kg) and connected to a ventilator via tracheal intubation. The heart was exposed via a left side limited thoracotomy and the middle of the left anterior descending artery (LAD) ligated with an 8-0 silk suture. At 60 min after induction of MI, mice were randomized to receive intramyocardial injection of (1) PBS (MI group, *n*=12); (2) 1.0×10^6^ BM-MSCs (BM-MSC group, *n*=13); or (3) 1.0×10^6^ Rap1^−/−^-BM-MSCs (Rap1^−/−^-MSC group, *n*=12) at four sites in the border of the infarct area. Another group of mice (*n*=10) underwent thoracotomy without LAD ligation and served as controls (Control group).

### Hemodynamic assessment

At 4 weeks post-cell transplantation, mice were anesthetized and mechanically ventilated as described above. A 1.2-F pressure–volume conductance catheter connected to an ADVantage PV-Loop system (Scisence Inc., Ontario, Canada) for data acquisition was inserted into the LV cavity through the right carotid artery. Hemodynamic parameters, including LVESP and dp/dtmax, were recorded and analyzed using the LabScribe software (Scisence Inc.). The slope of the ESPVR was measured to evaluate heart function.

### Assessment of fibrosis and apoptosis

Following hemodynamic assessment, mice were killed for histological and immunohistochemical study. Hearts were quickly taken out, washed with cold PBS and fixed in formalin for 24 h. Hearts were then embedded in paraffin and cut into 5-*μ*m sections from the apex, mid-LV. Fibrosis in the different experimental groups was analyzed using a Masson’s Trichrome Stain Kit (HT15, Sigma, St. Louis, MO, USA). The percentage infarct size was calculated as (fibrosis area/total LVA)×100%. Apoptosis was evaluated with TUNEL staining as previously described.

### Immunohistochemical staining

Immunohistochemical staining was performed according to the standard protocol. Following incubation with 5% bovine serum albumin for 30 min, heart sections or cells were stained with primary antibody and incubated overnight at 4 °C with a 1 : 100 dilution. The antibodies were anti-Troponin (SC-8121, Santa Cruz), anti-*α*-SMA (SC-53142, Santa Cruz), anti-CD31 (SC-31054, Santa Cruz) and anti-CD45 (SC-25590, Santa Cruz). Sections were incubated with PBS instead of the primary antibody to serve as a negative control. Then, the second antibody with FITC-conjugated anti-mouse IgG (1 : 1000), anti-rat IgG (1 : 1000) or anti-goat IgG (1 : 1000) was added and incubated for 1 h at room temperature. Finally, heart sections were washed with PBS twice and mounted with 4, 6-diamidino-2-phenylindole (DAPI) to stain the nucleus. Six mice from each group were analyzed; five sections were randomly collected from each mouse and then analyzed with a deconvoluted fluorescent microscope and Image J software (National Institutes of Health, Bethesda, MD, USA).

### Statistical analysis

Data are expressed as mean±S.D. The significant differences between groups were analyzed with unpaired Student’s *t*-test for two groups or one-way ANOVA, followed by Bonferroni test for more than two groups. A value of *P*<0.05 was considered statistically significant.

## Abbreviations

BM: bone marrow
CdM: conditioned medium
ESPVR: end systolic pressure volume relationship
IL-6: interleukin-6
LVESP: left ventricle end systolic pressure
MCP-1: monocyte chemotactic protein 1
MI: myocardial infarction
MSCs: mensenchymal stem cells
NCMCs: neonatal cardiomyocytes
NF-*κ*B: nuclear factor-kappaB
TNF-*α*: tumor necrosis factor-*α* (TNF-*α*)
